# Recovery Following Peer and Text Messaging Support After Discharge From Acute Psychiatric Care in Edmonton, Alberta: Controlled Observational Study

**DOI:** 10.2196/27137

**Published:** 2021-09-03

**Authors:** Reham Shalaby, Marianne Hrabok, Pamela Spurvey, Rabab M Abou El-Magd, Michelle Knox, Rebecca Rude, Wesley Vuong, Shireen Surood, Liana Urichuk, Mark Snaterse, Andrew J Greenshaw, Xin-Min Li, Vincent Israel Opoku Agyapong

**Affiliations:** 1 Department of Psychiatry University of Alberta Edmonton, AB Canada; 2 Cumming School of Medicine University of Calgary Calgary, AB Canada; 3 Alberta Health Services Addiction and Mental Health Edmonton, AB Canada

**Keywords:** peer support, recovery, controlled observational study, inpatients, mental health, supportive text messages

## Abstract

**Background:**

Peer support is an emotional, social, and practical help provided by nonprofessionals to assist others in sustaining health behaviors. Peer support is valued in recovery-oriented models of mental health and is becoming increasingly implemented at the organizational level. Text messaging is a relatively low-cost, high-impact, and easily scalable program that uses existing technology, is devoid of geographic barriers, and is easily accessible to end users.

**Objective:**

This study aims to evaluate the effectiveness of an innovative peer support system plus a supportive text messaging program on the recovery of discharged patients from acute psychiatric care.

**Methods:**

This prospective, rater blinded, controlled observational study included 181 patients who were discharged from acute psychiatric care. Patients were randomized to one of four conditions: treatment as usual (follow-up care), daily supportive text messages only, peer support only, or peer support plus daily supportive text messages. A standardized self-report measure of recovery (Recovery Assessment Scale [RAS]) was completed at baseline, 6 weeks, 3 months, and 6 months. Descriptive analysis, one-way analysis of variance, and repeated measures multivariate analysis of covariance were used to examine the changes in the RAS among the study groups and over the follow-up time points.

**Results:**

A total of 65 patients completed the assessments at each time point. For the overall sample, higher scores were found for the peer support plus text message condition compared with the text message only and treatment as usual condition on several scales (ie, willingness to ask for help and personal confidence and hope) and total score on the RAS, after 6 months of intervention.

**Conclusions:**

Peer support plus supportive text messaging seems to result in improved recovery compared with other interventions. It may be advisable to incorporate the two interventions as part of routine practice for patients with psychiatric disorders upon hospital discharge.

## Introduction

### Background

Peer support is emotional, social, and practical help provided by nonprofessionals to assist others in sustaining health behaviors [1]. The supporters share a similar condition as patients, successfully manage their conditions, and have received training to provide support [2]. Peer support may include activities such as advocacy, connecting resources, and experiential sharing [3]. Peer support is consistent with the recovery paradigm in mental health [4], and the purported mechanisms through which it functions [2] include knowledge sharing, modeling adaptive coping strategies, social comparison, and enhancing social support. Moreover, peer support systems can serve as an entry point into the health care system for *hardly reached* individuals and can provide support for those who would otherwise not engage in treatment [1]. Peer support may also offer benefits to peer supporters by enhancing feelings of competence and meaning [2].

Peer support is valued in recovery-oriented models [4] of mental health and is becoming increasingly implemented organizationally [5,6]. A review reported positive outcomes, including lower inpatient service use, better relationships with providers, and increased engagement [7]. However, a rigorous evaluation of randomized controlled trials (RCTs) [8] of peer support studies reported that outcomes were mixed and often nonsignificant. In their review, the authors noted a high degree of bias and methodological limitations in the studies, including inconsistent training for peer support workers (PSWs), lack of randomization of patients, and lack of blinding of outcome assessors, and concluded that “peer support programmes should be implemented within the context of high-quality research projects wherever possible.”

The existing literature suggests that peer support is valuable, but a more rigorous methodology to evaluate peer support program outcomes is needed. This study used an RCT design to evaluate a peer support model, which incorporates, as an innovative adjunct intervention, daily supportive text messages (TxM), provision of consistent training to PSWs, adopting blindness of the assessor, and randomization of the allocated patients.

Text messaging is a relatively low-cost, high-impact, and easily scalable program that uses existing technology, is devoid of geographic barriers, and is easily accessible to end users. Several RCTs have shown significant decreases in symptomatology in psychiatric conditions after the implementation of text messaging [9,10] and high rates of satisfaction among end users [11]. During the COVID-19 pandemic, supportive text messaging has been effective in decreasing symptomatology at the general population level [12]. Incorporating such services as a standard for patients upon their discharge from acute care may significantly improve the clinical and nonclinical outcomes for these patients and the health care system.

### Study Aim

The overall aim of this project is to evaluate the effectiveness of innovative peer support and supportive text messaging systems as either stand-alone or combined interventions in addition to the usual treatment for patients discharged from acute care.

## Methods

### Study Design

Although the initial intention was to conduct an RCT [13], subject recruitment and treatment arm allocation issues necessitated an early planned transition to a controlled observational study, as described in the following sections. Participants were recruited from June 2019 to February 2020 and were randomized into one of four conditions: (1) PSW only, (2) TxM only, (3) PSW plus TxM condition (PSW+TxM), and (4) treatment as usual (TAU). Written consent was obtained and no incentives were provided.

Initial randomization was performed by an independent statistician using the block randomization method. The generated codes were sent securely to the study coordinator to assign the recruited patients across the four arms of the study treatment groups. Participants were asked at the beginning of the interview to not reveal their treatment allocation to the researcher who would facilitate the follow-up assessments. The study database was updated by the study coordinator upon recruitment. Randomization codes were kept secured on a password-protected computer. To further maintain the blindness, the researcher conducting follow-up assessments was not granted access to the database that contained the randomization code*.*

The study was approved by the Health Ethics Research Board of the University of Alberta (reference number Pro00078427) and operational approval from Alberta Health Services, the regional health authority. Written informed consent was obtained from all the patients. The study was registered with ClinicalTrials.gov (trial registration: NCT03404882). In relation to the design change to a controlled observational study, the amendments to the study protocol [13] are now reflected in a revised registered trial protocol for NCT0340488.

### Study Locations

The study was conducted at 5 acute psychiatric care units in Edmonton, Alberta, Canada. Patients were invited to participate in the study before their discharge.

### Participants

#### Patients

The inclusion criteria were as follows: mental health condition (mood or psychotic disorder), imminent discharge from acute care, 18 to 65 years of age, able to provide written consent, and a mobile handset capable of receiving text messages. The exclusion criteria were as follows: inability to read the text messages from a mobile device, an addiction disorder without a mental health diagnosis, receiving PSW service before the study, or inability to commit to a sixth-month follow-up of the study.

#### Peer Support Workers

PSWs in this study were employed by Alberta Health Services Edmonton Zone Addiction and Mental Health Services after receiving 2 weeks of formal training. The PSW training program was designed by Cusick [14] and covered 13 domains: recovery and peer support; the history of recovery movement; worldview and culture; self-determination; trauma-informed care; boundaries and limits; communication and connection; the social determinants of health; impact of prejudice, discrimination, and stigma; grief and loss; crisis and recovery; goal planning; and self-care. In alignment with the literature [15], matching PSWs with our patients with respect to their baseline mental health conditions was not a criterion for assigning candidate patients to a PSW.

#### Demographic Characteristics of PSWs

A total of 8 dedicated PSWs were enrolled in this study (1 male and 7 females). They are employed by Alberta Health Services and occupy different positions in different health care settings within the Addiction and Mental Health portfolio. As described earlier, PSWs were not matched to our patients based on their mental health conditions, and so the mental health diagnosis of the PSWs in this study was not ascertained.

### Treatment Interventions

In the PSW-only condition, a PSW met physically or virtually with the patients up to eight times over a 6-month period to offer mental health support. In the TxM-only condition, TxM were received without additional PSW intervention. In the PSW+TxM condition, participants were offered PSW services along with daily TxM. In the control arm, conventional follow-up appointments with community providers were offered but neither PSW nor TxM were provided.

1. Peer support service: patients in the PSW-only and PSW+TxM arms of the study were assigned PSWs who visited them (one to one) at the hospitals to introduce themselves and build rapport before patients were discharged into the community. PSWs visited the participants up to eight times over a 6-month period (mean 3 visits, SD 2.5). They offered the opportunity for interactive phone calls and/or texts between themselves and patients for 6 months. Phone calls or virtual meetings were offered to replace face-to-face meetings during the COVID-19 pandemic. Patients continued to receive usual community clinic or program treatments.
2. Text4Support: this is a daily supportive text message service conceived and designed by a group of psychiatrists, psychologists, mental health therapists, and patients based on cognitive behavioral therapy principles [16]. A bank of messages was generated and included different text message programs tailored for the following eight mental health domains: depression, anxiety, psychotic disorders, substance use disorders, bipolar disorder, adjustment disorders, attention-deficit or hyperactivity disorder, and general well-being. About 80% of the messages in all eight message banks had similar general well-being content; the remaining 20% targeted diagnosis-specific symptoms. Patients were enrolled by the research team to receive an assigned message bank based on their primary diagnosis by linking their phone number to the message bank through a web-based application (software). Patients in the automated TxM-only and PSW+TxM arms of this study received automated messages at 10 AM Mountain Time. Examples of these messages include the following:
  - Notice the good things going on in your life right now. Often, we do not notice the good but taking a moment to do so can uplift you. (General well-being)
  - When we are anxious, our thoughts often focus on future “danger.” Shift your attention to the present. What is happening right now? (Anxiety)
  - Self-monitoring helps you identify and distinguish between normal changes in mood and mood swings that are problematic. (Bipolar disorder)
  - Try talking quietly back to voices. Tell them they are wrong. Using the vocal part of the brain can reduce the intensity of voices. (Psychosis)

### Outcomes

Participants completed measures at baseline, 6 weeks, 3 months, and 6 months. The primary outcome measure for this study was recovery, as assessed by the Recovery Assessment Scale (RAS [17]), a standardized instrument with strong psychometric properties, including high internal consistency (α=.93), test-retest reliability (*r*=0.88), and concurrent validity [18]. Furthermore, the scale is sensitive to changes over time [19]. This 24-item scale provides self-reported recovery ratings on a 5-point Likert scale (strongly disagree=1, disagree=2, not sure=3, agree=4, and strongly agree=5). The RAS subscales include five factors: (1) *personal confidence and hope* (response range 9-45); (2) *willingness to ask for help* (response range 4-20); (3) *goal and success orientation* (response range 3-15); (4) *reliance on others* (response range 5-25); and (5) *no domination by symptoms* (response 3-15). The Cronbach α coefficients for the five subscales range from 0.74 to 0.87, and the total score is positively associated with quality of life and empowerment, whereas it is inversely associated with symptoms [20]. Total scores (raw scores) were calculated for the composite RAS and for each of the five subscales and were used in the analysis of this study [19,21-23].

### Sample Size

Consistent with the idea that this was a pilot study without an empirically established effect size available to aid in power and sample size calculations, the targeted sample size of 180 participants was based on existing operational resources [24].

### Data Analysis

The analysis was conducted using SPSS version 20 (IBM Corp, 2011) [25]. Initially, we aimed to use intention-to-treat analysis, whereby patient data were analyzed according to their original assigned groups, regardless of the time spent in the study. However, after randomization and due to clinical logistic reasons, a significant number of patients did not receive access to the PSW service in the two intervention arms of the PSW. As stated earlier, a strategic decision was made to adapt the protocol to a controlled observational study and to change the analysis approach to as-treated, rather than intention-to-treat, to maximize the investigational value of the study without compromising or biasing outcomes.

Baseline data, including sociodemographic (age group, gender, ethnicity, education level, employment status, and relationship) and clinical characteristics (primary diagnosis and RAS five factors), were analyzed to assess between-group differences across the four arms of the study (PSW-only condition, TxM-only condition, PSW plus TxM condition, and TAU condition). The analysis was conducted using chi-square and one-factor analysis of variance (ANOVA) for categorical and continuous variables, respectively.

Age categories were generated in accordance with the quartile distribution of the age-in-years variable. RAS factors were analyzed to assess cluster differences among the four study arms across the four periods of the study, using mean and SD. A one-factor ANOVA followed by Tukey post hoc test was performed to assess the statistical differences between the study arms and corresponding mean scores on each RAS factor for all the participants who completed the follow-up assessment at any designated follow-up time point. Welch F and Games-Howell post hoc tests were performed when there was evidence of a violation of the homogeneity of variance assumption. For participants who completed assessments at all the four time points, a repeated measures multivariate analysis of covariance (MANCOVA) was used to assess the impact of the four arms of the study on participants’ scores of the RAS five factors across the three time points (6 weeks, 3 months, and 6 months follow-up), while controlling for baseline scores. With regard to MANCOVA post hoc analysis, Bonferroni corrections were used to control for multiple comparison error rate changes for post hoc pairwise analyses.

CIs and *P* values were used in reporting. Cases with missing values of more than one individual response per factor were excluded from the analysis. The two-tailed α-level criterion for statistical significance was set at *P*≤.05.

## Results

### Participant Flowchart

**Figure 1:**
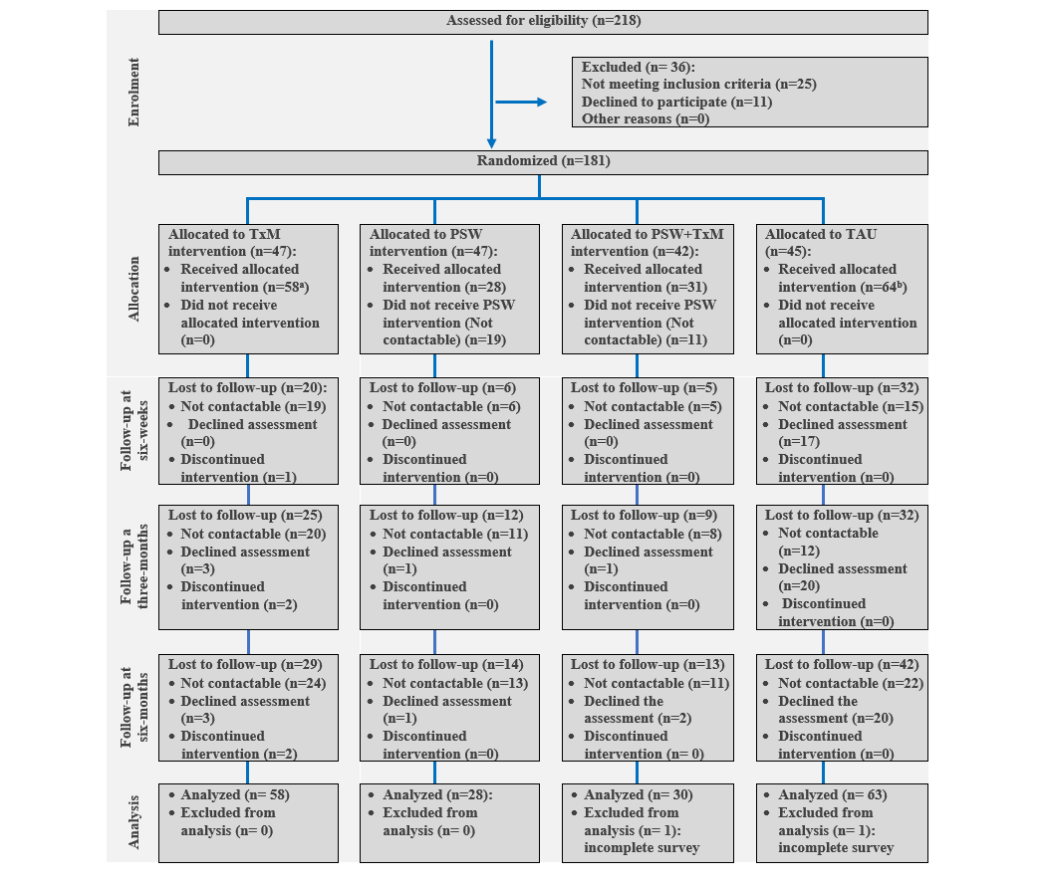
Study flow chart.

The study flowchart is presented in Figure 1. A total of 181 patients were recruited and randomized into four study arms (n=43-47 per condition). At 6 weeks, 64.6% (117/181) of patients responded to the RAS survey, whereas 56.9% (103/181) of patients responded at 3 months, and 45.9% (83/181) of patients responded to the 6-month survey, yielding an aggregate time point response range between 45.9% and 64.6%.

Some patients were randomized to receive the PSW intervention with or without TxM support but did not receive PSW interventions for several reasons, including subsequent noninterest in receiving visits from PSWs and failure of PSWs to contact (Figure 1). Some of these participants continued to receive only TxM support or only TAU but attended follow-up assessments. Given the relatively small sample size of our study and the overarching objective of assessing the actual effects of the interventions, we adapted our study analysis plan to simply assess outcome data with regard to either the TAU or TxM support-only groups, reflecting the service they actually received.

### Participant Characteristics

In terms of demographic and clinical characteristics (Table 1), the overall gender balance was fairly even with 56.9% (103/181) identifying as female, 27.1% (49/181) in the range of 50-65 years of age, 69.1% (125/181) identifying as White, 55.9% (100/179) achieved a postsecondary education level, 69.4% (125/180) are unemployed, 50.8% (84/179) are single, and 50.8% (92/181) were admitted for depression and/or anxiety.

Chi-square and ANOVA results indicated that participants in the four treatment arms did not significantly differ in terms of sociodemographic and clinical parameters at baseline (*χ^2^*_3_=2.7 to *χ^2^*_3_=6.8, *P=*.08 to *P*=.91; *F*_3,175_=0.39-1.60, *P*=.19 to *P*=.76).

**Table 1:** Demographic and clinical characteristics of study participants in the study arms.

Demographic and clinical variables			TxM^a^ only, n (%)		PSW^b^ only, n (%)		PSW+TxM, n (%)		TAU^c^, n (%)		Total, n (%)		Chi-square (*df*)			*P* value	
**Gender**														6.8 (3)			.08
	Male	18 (31)		14 (50)		18 (58.1)		28 (43.8)		78 (43.1)							
	Female	40 (69)		14 (50)		13 (41.9)		36 (56.2)		103 (56.9)							
**Age groups (years)**														10.9 (9)			.28
	18-30	17 (29.3)		9 (32.1)		8 (25.8)		14 (21.9)		48 (26.5)							
	31-40	9 (15.5)		6 (21.4)		6 (19.4)		19 (29.7)		40 (22.1)							
	41-50	11 (19)		10 (35.7)		8 (25.8)		15 (23.4)		44 (24.3)							
	50-65	21 (36.2)		3 (10.7)		9 (29.0)		16 (25.0)		49 (27.1)							
**Ethnicity**														5.3 (6)			.51
	Indigenous	5 (8.6)		4 (14.3)		4 (12.9)		12 (18.8)		25 (13.8)							
	White	46 (79.3)		18 (64.3)		20 (64.5)		41 (64.1)		125 (69.1)							
	Other	7 (12.1)		6 (21.4)		7 (22.6)		11 (17.2)		31 (17.1)							
**Educational level**														4.8 (6)			.58
	Less than high school	6 (10.3)		7 (25)		5 (16.1)		11 (17.7)		29 (16.2)							
	High school degree or equivalent	19 (32.8)		7 (25)		6 (19.4)		18 (29.0)		50 (27.9)							
	Above high school education	33 (56.9)		14 (50)		20 (64.5)		33 (53.2)		100 (55.9)							
**Employment status**														2.7 (3)			.45
	Employed	16 (27.6)		7 (25)		8 (25.8)		24 (38.1)		55 (30.6)							
	Unemployed	42 (72.4)		21 (75)		23 (74.2)		39 (61.9)		125 (69.4)							
**Relationship**														5.1 (6)			.53
	Married, common law, or in relationships	11 (19)		11 (39.3)		10 (33.3)		16 (25.4)		48 (26.8)							
	Single	30 (51.7)		12 (42.9)		12 (40.0)		30 (47.6)		84 (46.9)							
	Divorced, separated, or widowed	17 (29.3)		5 (17.9)		8 (26.7)		17 (27.0)		47 (26.3)							
**Admitting diagnosis**														6.6 (6)			.37
	Depression and/or anxiety	27 (46.6)		14 (50)		16 (51.6)		35 (54.7)		92 (50.8)							
	Bipolar disorder	21 (36.2)		10 (35.7)		10 (32.3)		12 (18.8)		53 (29.3)							
	Psychotic disorder	10 (17.2)		4 (14.3)		5 (16.1)		17 (26.6)		36 (19.9)							

^a^TxM: supportive text messages.

^b^PSW: peer support worker.

^c^TAU: treatment as usual.

### Study Outcome

For the overall sample (with variable N for each time point as shown in Table 2), ANOVA revealed a statistically significant difference between- and within-groups for scores of *personal confidence and hope* factor at the 3-month follow-up (*F*_3,99_=3.35; *P*=.02); *willingness to ask for help* factor at the 6-month follow-up (*F*_3,79_=3.89; *P*=.01); and total recovery score at the 6-month follow-up. Tukey post hoc tests revealed a significantly higher mean of the *personal confidence and hope* factor at 3 months in the PSW+TxM arm than in the TxM-only arm (mean difference 5.09, 95% CI 0.41-9.77; *P*=.03) and TAU arm (mean difference 5.09, 95% CI 0.38-9.8; *P*=.03). In addition, a significantly higher mean for *willingness to ask for help* was detected for the PSW+TxM arm than for the TxM-only arm (mean difference 1.87, 95% CI 0.22-3.51; *P*=.02). Similarly, for the total recovery score, the PSW+TxM arm had a significantly higher mean than the TxM-only condition (mean difference 11.78, 95% CI 3.08-20.48; *P*<.01).

For patients who completed the RAS (n=65) at all four time points (Table 3; PSW: n*=*13; TxM: n*=*19; PSW+TxM: n*=*12; TAU: n*=*19), we performed repeated measures MANCOVA, with treatment intervention as the independent variable, RAS score and subscores as the dependent variables, and baseline scores as covariates. With sphericity accepted, tests of within-subject effects indicated that neither time (*F*_10,47_=1.47; *P*=.18; ηp2=0.24) nor the interaction of time and PSW (*F*_10,47_=1.20; *P*=.31; ηp2=0.20), time and TxM (*F*_10,47_=.48; *P*=.89; ηp2=0.09), or time and PSW+TxM (*F*_10,47_=1.24; *P*=.29; ηp2=0.21) significantly predicted RAS subscores and total scores. However, tests of between-subject effects indicated that the interaction between PSW and TxM was predictive of differences in scores on only the goal and success subscale (*F*_1,63_=4.37; *P*=.04; ηp2=0.072) and reliance on other subscales (*F*_1,63_=6.24; *P*=.02; ηp2=0.10).

**Table 2:** Mean and SD of the Recovery Assessment Scale total score and factor scores by study condition for patients who completed assessments at any of the four time points.

RAS^a^ score and time			TxM^b^ only					PSW^c^ only					PSW+TxM					TAU^d^		
			Patients, n (%)		Value, mean (SD)		Patients, n (%)			Value, mean (SD)		Patients, n (%)			Value, mean (SD)		Patients, n (%)			Value, mean (SD)
**Personal confidence and hope**																				
	Baseline (n=179)	58 (32.4)		33.4 (6.67)		28 (15.6)			34.5 (7.23)		30 (16.8)			35.6 (8.69)		63 (35.2)			32.1 (8.46)	
	6 weeks (n=117)	37 (31.6)		32.6 (5.91)		22 (18.8)			33.6 (5.53)		26 (22.2)			35.6 (6.26)		32 (27.4)			32.0 (6.5)	
	3 months (n=103)	33 (32)		31.1 (5.81)		16 (15.5)			32.4 (6.57)		22 (21.4)			31.1 (7.57)		32 (31.1)			31.1 (7.57)	
	6 months (n=83)	29 (34.9)		31.5 (6.37)		14 (16.9)			34.0 (5.82)		18 (21.7)			35.9 (3.65)		22 (26.5)			32.3 (6.83)	
**Goal and success**																				
	Baseline (n=179)	58 (32.4)		16.5 (2.72)		28 (15.6)			16.7 (3.22)		30 (16.8)			17.0 (3.12)		63 (35.2)			15.8 (3.13)	
	6 weeks (n=117)	37 (31.6)		15.6 (2.44)		22 (18.8)			15.4 (3.0)		26 (22.2)			17.0 (2.43)		32 (27.4)			15.2 (3.21)	
	3 months (n=103)	33 (32)		15.0 (3.27)		16 (15.5)			14.9 (2.73)		22 (21.4)			16.6 (2.48)		32 (31.1)			14.8 (3.4)	
	6 months (n=83)	29 (34.9)		15.0 (3.24)		14 (16.9)			15.6 (2.95)		18 (21.7)			17.1 (2.4)		22 (26.5)			15.5 (2.7)	
**Willingness to ask for help**																				
	Baseline (n=179)	58 (32.4)		11.5 (2.54)		28 (15.6)			11.8 (2.7)		30 (16.8)			12.8 (2.08)		63 (35.2)			11.8 (2.85)	
	6 weeks (n=117)	37 (31.6)		11.2 (2.52)		22 (18.8)			11.5 (1.92)		26 (22.2)			12.4 (1.65)		32 (27.4)			11.8 (1.83)	
	3 months (n=103)	33 (32)		11.3 (2.27)		16 (15.5)			12.0 (2.16)		22 (21.4)			12.4 (2.08)		32 (31.1)			11.9 (2.74)	
	6 months (n=83)	29 (34.9)		11.0 (2.6)		14 (16.9)			12.7 (1.68)		18 (21.7)			12.8 (1.79)		22 (26.5)			11.7 (1.75)	
**Reliance on others**																				
	Baseline (n=179)	58 (32.4)		20.3 (3.72)		28 (15.6)			21.5 (3.29)		30 (16.8)			21.3 (2.71)		63 (35.2)			20.4 (3.39)	
	6 weeks (n=117)	37 (31.6)		19.4 (3.51)		22 (18.8)			20.0 (2.77)		26 (22.2)			20.7 (2.17)		32 (27.4)			19.5 (3.65)	
	3 months (n=103)	33 (32)		19.7 (2.88)		16 (15.5)			20.5 (2.78)		22 (21.4)			21.6 (2.32)		32 (31.1)			20.3 (3.13)	
	6 months (n=83)	29 (34.9)		19.5 (2.89)		14 (16.9)			21.0 (2.86)		18 (21.7)			21.2 (2.71)		22 (26.5)			20.5 (3.23)	
**No domination by symptoms**																				
	Baseline (n=179)	58 (32.4)		9.2 (3.12)		28 (15.6)			9.7 (3.44)		30 (16.8)			9.9 (3.8)		63 (35.2)			9.3 (3.36)	
	6 weeks (n=117)	37 (31.6)		9.5 (2.45)		22 (18.8)			9.2 (2.74)		26 (22.2)			10.1 (2.41)		32 (27.4)			9.8 (2.92)	
	3 months (n=103)	33 (32)		9.5 (3.04)		16 (15.5)			9.1 (3.2)		22 (21.4)			10.2 (2.92)		32 (31.1)			9.2 (3.5)	
	6 months (n=83)	29 (34.9)		9.3 (3.19)		14 (16.9)			10.7 (2.09)		18 (21.7)			11.0 (2.17)		22 (26.5)			9.3 (3.14)	
**RAS total**																				
	Baseline (n=179)	58 (32.4)		90.9 (14.16)		28 (15.6)			94.2 (17.01)		30 (16.8)			96.0 (17.5)		63 (35.2)			89.4 (17.83)	
	6 weeks (n=117)	37 (31.6)		88.2 (11.6)		22 (18.8)			89.6 (13.46)		26 (22.2)			95.9 (12.86)		32 (27.4)			88.2 (14.09)	
	3 months (n=103)	33 (32)		86.6 (12.25)		16 (15.5)			88.9 (13.69)		22 (21.4)			97.0 (12.73)		32 (31.1)			87.3 (17.73)	
	6 months (n=83)	29 (34.9)		86.3 (13.88)		14 (16.9)			94.1 (13.33)		18 (21.7)			98.1 (8.48)		22 (26.5)			89.3 (15.03)	

^a^RAS: Recovery Assessment Scale.

^b^TxM: supportive text messages.

^c^PSW: peer support worker.

^d^TAU: treatment as usual.

**Table 3:** Mean and SD of the Recovery Assessment Scale total score and factor scores by study condition for patients who completed assessments at all four time points.

RAS^a^ score and time			TxM^b^ only (n=19), mean (SD)		PSW^c^ only (n=13), mean (SD)		PSW+TxM (n=13), mean (SD)		TAU^d^ (n=20), mean (SD)
**Personal confidence and hope**									
	Baseline	35.00 (6.63)		35.77 (7.50)		34.69 (10.37)		32.1 (8.23)	
	6 weeks	32.32 (6.19)		33.08 (5.55)		35.15 (6.31)		32.00 (6.16)	
	3 months	32.32 (5.14)		32.31 (7.32)		35.77 (5.34)		31.35 (7.37)	
	6 months	33.11 (6.04)		33.92 (6.05)		35.77 (3.59)		32.30 (6.87)	
**Goal and success**									
	Baseline	16.47 (2.93)		16.85 (3.41)		16.54 (4.18)		15.8 (2.82)	
	6 weeks	15.26 (2.81)		14.85 (3.26)		16.85 (2.44)		15.55 (3.33)	
	3 months	15.32 (3.76)		14.77 (2.77)		16.54 (2.33)		14.70 (3.20)	
	6 months	15.11 (3.40)		15.62 (3.07)		17.38 (2.47)		15.80 (2.97)	
**Willingness to ask for help**									
	Baseline	10.79 (3.46)		12.23 (3.35)		13.08 (1.80)		11.4 (3.10)	
	6 weeks	11.11 (2.71)		11.69 (2.06)		12.92 (1.38)		11.95 (1.93)	
	3 months	11.21 (2.72)		12.23 (2.24)		12.23 (1.92)		11.80 (2.57)	
	6 months	11.16 (2.65)		12.77 (1.74)		12.92 (1.98)		11.65 (1.76)	
**Reliance on others**									
	Baseline	20.3 (3.30)		22.08 (3.17)		21.77 (.59)		20.25 (3.13)	
	6 weeks	19.74 (3.35)		20.31 (2.66)		21.15 (1.99)		20.15 (3.18)	
	3 months	19.42 (3.19)		20.77 (3.00)		21.92 (2.02)		20.55 (2.76)	
	6 months	19.6 (3.25)		21.8 (2.96)		21.85 (1.95)		20.60 (2.74)	
**No domination by symptoms**									
	Baseline	10.95 (3.26)		10.46 (3.07)		9.38 (3.86)		9.90 (2.73)	
	6 weeks	9.53 (2.86)		9.23 (2.95)		9.62 (2.36)		10.05 (3.02)	
	3 months	10.74 (2.54)		9.54 (3.31)		9.62 (2.82)		9.45 (3.38)	
	6 months	10.47 (3.10)		10.54 (2.07)		11.00 (2.12)		9.45 (3.20)	
**RAS total**									
	Baseline	93.84 (15.32)		97.38 (16.98)		95.46 (20.20)		89.45 (15.46)	
	6 weeks	87.95 (12.35)		89.15 (13.74)		95.69 (12.45)		89.70 (14.19)	
	3 months	89.00 (12.83)		89.62 (15.03)		96.08 (12.05)		87.85 (17.01)	
	6 months	89.47 (14.59)		93.92 (13.86)		98.92 (8.37)		89.55 (14.45)	

^a^RAS: Recovery Assessment Scale.

^b^TxM: supportive text messages.

^c^PSW: peer support worker.

^d^TAU: treatment as usual.

## Discussion

### Principal Findings

To our knowledge, this is the first study to evaluate the effects of an innovative peer support program that incorporates supportive text messaging on recovery outcomes in patients discharged from acute psychiatric care under optimum controlled observational study conditions. An ongoing RCT in the United Kingdom is examining the effects of peer worker support for patients discharged from acute care in comparison with patients receiving TAU [26]; however, that study did not include a supportive eHealth component such as the text messaging support included in our controlled observational study.

Despite the relatively small sample size in our study, patients in the PSW+TxM group had notably higher recovery scores compared with those receiving either TxM-only or TAU. The study measures that were included provide potentially important information regarding the mechanisms of change enacted by peer support. For example, although the mechanism of change in peer support is unclear, our results suggest that peer support may influence personal confidence and hope as well as enhance the ability of patients to ask for help.

It is notable in this study that most patients who refused to complete the follow-up assessment were in the TAU group (n=20), compared with the other groups (maximum for other groups=3). This may be explained by a lack of interest. When patients receive no actual intervention, they become less motivated to provide feedback related to the research under study. Dropout figures were the highest among the patients who were assigned to the PSW service [27]. Some patients stated that they preferred to *control* their path of recovery after hospital discharge. Others were not suitable candidates for this service during this initial vulnerable postdischarge period, as assessed by the PSW; many PSW-allocated patients proved hard to reach, and in such cases, PSW follow-up is usually terminated or at least significantly interrupted [27].

A recent systematic review explored different interventions, including peer support, to improve the successful transition for discharge from acute mental health inpatient care to the community [28]. In one Australian study, 38 patients achieved recovery and wellness (particularly clinical and functional recovery) after receiving peer support for 6-8 weeks, which is consistent with our results [29]. In another Australian study, 49 patients receiving peer support, as supportive packages for 8-12 hours for 1-2 weeks, reported that the intervention solidified their recovery and improved their self-confidence [30]. In contrast, in a UK study, in which 23 patients received peer support for 4 weeks and 23 were in the TAU group, there was no evidence of a significant difference between the two groups regarding hopefulness [27]. Unlike the three studies reviewed in the systematic review [28], our study findings indicate the relative impact of combined delivery of PSW+TxM compared with peer support alone, which may explain the discrepancy noted with the UK study [27].

Other studies have provided peer support to discharged patients either alone or alongside other interventions, such as environmental support or brief intervention (eg, interactive behavior change technology). However, those studies assessed outcomes other than patient recovery or reported mixed findings [31,32]. The positive effect of the combined delivery of PSW+TxM observed in our study included TxM provided to the patients that were tailored according to their diagnosis. Previous studies have reported positive benefits of receiving daily TxM in the context of mental health and addiction. For example, patients with depression alone or comorbid with alcohol use disorder reported the effectiveness of texting service on symptom recovery in terms of better management of depression and anxiety and perceived better overall mental well-being [33]. In addition, a longer time to first drink was reported after receiving TxM for 3 months and was maintained for up to 6 months [11,34]. Multiple advantages have been reported when using texting services in patients with psychotic disorders, including better medication adherence, improved clinical and functional symptoms, effective symptom monitoring, and high acceptability by end users [35-37].

Studies examining the effect of a supportive texting service for patients discharged from acute care are rare. A recent systematic review focusing on web- and mobile phone–based texting in mental health [38] reported some studies that offered texting services to patients on hospital or emergency discharge with different mental health conditions, including alcohol use disorder, bulimia nervosa, and suicide. Each of these studies reported positive outcomes, including decreased binge drinking, reduced remission rates, and achieved feasibility and acceptability by patients who attempted suicide. In contrast, our study did not produce more favorable recovery outcomes for patients who received only TxM along with TAU. This contrasts with previous findings that patients with major depressive disorder who received daily TxM in addition to usual treatment had significantly fewer depressive symptoms and improved quality of life compared with TAU [9,10]. It is interesting to note that those previous RCTs used the Beck Inventory Score changes at 3 months from baseline as the outcome measure, while this study assessed recovery outcomes using the RAS. The fact that patients in our study received text messages once daily, whereas patients in two previous RCTs [9,10] received twice daily text messages may also be related to differences in these study outcomes.

A growing body of evidence supports the paradigm of integration of health care services through multidisciplinary intervention or support. This appears to have a particularly high potential impact when patients are facing multiple and complex needs that can progress to severe forms of mental illness [39,40].

The results of this controlled observational pilot study have the potential to signal an important direction for future studies to incorporate these integration goals into peer support programs.

### Study Limitations

Our study had several important limitations. For instance, only the RAS recovery outcome measure is reported in this study, and it is important to examine the effects of peer support and daily supportive text messaging on multiple outcomes, including quality of life, symptomatology, and health care use and functional outcomes, such as employment [13]. In addition, the RAS is a self-report outcome questionnaire and is therefore subject to social desirability and another weakness in this study. For future studies, it will be important to maximize adherence to self-reporting across the time points assessed. This can be achieved via incentives linked to completion.

Importantly, high dropout and/or nonservice provision rates for PSW among the study participants undermined the initial RCT design, thereby reducing the robustness of the study results. Consequently, to be able to access the actual impact of the interventions, we adopted a controlled observational study with a *to-treat analysis* rather than the original RCT plus an *intention-to-treat analysis*.

Although this study provided important preliminary information regarding the outcomes of peer support programs for patients discharged from acute care, the overall study sample and the individual group sizes were relatively small. Small sample sizes reduce study power and the sensitivity of studies to detect differences between treatment groups. A multicenter study with large sample sizes will be needed to validate the results of this study and to determine the actual effect size of the various interventions forming a part of this controlled observational study.

## Abbreviations

ANOVA: analysis of variance
MANCOVA: multivariate analysis of covariance
PSW: peer support worker
RAS: Recovery Assessment Scale
RCT: randomized controlled trial
TAU: treatment as usual
TxM: supportive text messages
